# Gender Differentiation of Indirect Self-Destructiveness in Drug Addicted Individuals (Indirect Self-Destructiveness in Addicted Women and Men)

**DOI:** 10.1007/s11126-019-09629-0

**Published:** 2019-03-05

**Authors:** Konstantinos Tsirigotis

**Affiliations:** Department of Psychology, The Jan Kochanowski University in Kielce, Piotrków Trybunalski Branch, 114/118 Słowackiego St, 97-300 Piotrków Trybunalski, Poland

**Keywords:** Women, Men, Psychoactive substances, Addiction, Gender differentiation, Indirect self-destructiveness

## Abstract

The use of psychoactive substances is considered to be a typical self-destructive behaviour with addiction itself regarded as one of the self-destructiveness forms. The aim of this work was to explore the gender differentiation of the indirect self-destructiveness syndrome (and its particular categories) in drug addicted individuals treated in drug addiction treatment centres. 172 drug addicted individuals (116 men and 56 women, M age = 23,5), ranged from 19 to 28 years, was recruited. In order to examine indirect self-destructiveness and its manifestations, the Polish version of the “Chronic Self-Destructiveness Scale” by Kelley (CS-DS) was administered. The statistical processing of scores used the Mann-Whitney U significance test. Women treated for drug addiction achieved significantly higher scores on indirect self-destructiveness: general score (*p* = 0.001), subscales of Transgression and Risk (p = 0.001), Personal and Social Neglects (*p* = 0.02), and Lack of Planfulness (*p* < 0.001). They scored lower on Poor Health Maintenance (*p* < 0.002) and Helplessness (p < 0.001). There is a need for specific, gender-adjusted manners of intervention and treatment in addicted women. Optimistically, after an addiction treatment, women cope and feel better psychologically and socially. They also care more about their health.

## Introduction

The human is not only a biological but also psychological and social being whereas psychoactive substance use/abuse/addiction is a complex psycho(patho)logical phenomenon resulting from interactions of biological, psychological and social or cultural factors. Among those factors, the biological sex, gender and social environment can be distinguished. Interactions of those factors determine differences in the course of addiction and treatment in women and men.

The use of psychoactive substances, is considered to be a typical self-destructive behaviour with addiction itself regarded as one of the self-destructiveness forms [1–4].

### Indirect Self-Destructiveness

A majority of authors usually consider “self-destructive behaviours” to be behaviours categorised as directly self-destructive: self-mutilation, self-inflicted injury and attempted or committed suicide [5–9]. There is a distinction between a direct and indirect threat and/or harm. It is important that indirectly self-destructive behaviours almost imperceptibly cause harmful side effects, although a great number of such behaviours are classified by most as being a (broader) standard or norm [10].

Chronic or indirect self-destructiveness is described as a generalised tendency towards undertaking behaviours that increase the probability of negative and reduce the probability of positive consequences for the subject [2]. Indirect self-destructiveness encompasses both taking and abandoning (not only commission but also omission of) particular actions. That concerns getting into dangerous and elevated-risk situations (active form) or neglecting one’s own safety or health (passive form). Indirect self-destructiveness is a self-destruction form characterised by an increased distance in time between an action and its consequence [4, 11]. Several categories of indirectly self-destructive behaviours are distinguished: transgression and risk, poor health maintenance, personal and social neglects, lack of planfulness, and helplessness and passiveness in the face of problems/difficulties [2, 4, 11]. Individuals mainly motivated by current emotional factors (“impulsive” ones) are more likely to carry out self-destructive acts in contrast to those motivated by more long-term cognitive considerations [2]. The capability of restraining impulses is closely connected with resistance to temptation. Yielding to temptation, present in the syndrome of self-destructive behaviours, serves many purposes such as pleasure, reduction of tension, self-stimulation, avoiding efforts and many others [4]. Moreover, according to many psychoanalytic theory assumptions, addiction is in fact rooted in unconscious self-destructive tendencies [12]. Not coincidentally, indirect-self destructiveness is called “slow” or “lingering” suicide.

### Psychoactive Substances and Indirect Self-Destructiveness

There are some internal and external risk factors of use/abuse/addiction common to women and men. Such psychological traits as high sensation seeking, impulsiveness and poor self-regulation are important risk factors of psychoactive substance use and abuse [13–16]. Such personality traits as neurobehavioural disinhibition/novelty seeking or high novelty seeking/low harm avoidance are good predictors of problems with psychoactive substances [16–21]. Those traits are components of transgression and risk being, as we could observe, an important category of indirect self-destructiveness.

Positive correlations were, for instance, found between indirect self-destructiveness and exhibiting risky behaviours, including drug/alcohol (ab)use, aggressive and/or criminal, risky sexual and irresponsible behaviours [22]. In addition, relationships were observed between drug (ab)use and direct self-destructiveness: suicidal ideation, suicide attempts and committed suicides [23].

### Women, Men and Psychoactive Substances

For some time now attempts have been made at distinguishing effects of psychoactive substance use/abuse/addiction among women and men. For instance, it was established that men use most psychoactive substances more than women [16].

Alcohol does not affect women and men to the same extent. As a result of metabolic differences, an intake of the same quantity of alcohol leads to a higher blood alcohol concentration in women than in men. Men drink more often and larger amounts, and more frequently abuse alcohol [24–28].

In turn, women more commonly than men use/abuse legal psychoactive substances, i.e. prescription drugs, while men more often use/abuse illegal substances like marijuana [25, 29–36].

Women’s stronger tendency to take medicines also for non-medical purposes was established in the population of individuals after suicide attempts as well: in that population women used medicines more often than men too [37].

### Women, Men and Substance Use Disorders

Women in the general population more commonly and willingly use health services and are more prone to report their complaints and symptoms to a health care professional who can diagnose them with psychological disorders in which medical therapy is indicated [25, 29, 30, 32].

Disturbances arising from psychoactive substance use/abuse/addiction occur more often in men [38–43].

Nevertheless, the time between drug initiation and problem use and the first attempt at treatment is shorter in women than in men. The progress of disease is faster in women, as is the occurrence of health and social problems resulting from substance abuse. The phenomenon, first established for alcohol, but concerning all substances, was called the “telescoping effect” and means that women progress through the landmark stages from the initial use to dependence at a faster rate than men [16, 33, 39, 44–50].

In addicted population, gender differences were found for direct self-destructiveness. Women more often think of suicide and more commonly attempt suicide, similarly to the general population [37]; in turn, the number of methods or means used by men attempting suicides is higher than in women [23]. Thus, it is the opposite of what is observed in the population of individuals attempting suicides but not using drugs [37].

The aim of this study was to explore the gender differentiation of the indirect self-destructiveness syndrome (and its particular categories) in drug addicted individuals treated in drug addiction treatment centres.

## Methods

The study is part of two more extensive projects (on indirect self-destructiveness and on drug addiction) and thus the applied methodology or certain other parts may be similar. Preliminary results of this project have been published earlier [51, 52].

### Participants

In order to meet the research objectives, a population of 172 drug addicted individuals (116 men and 56 women), charges of MONAR^1^^*^ type centres for drug treatment, was investigated.

The respondents’ age ranged from 19 to 28 years (mean age: 23.5); incomplete secondary and secondary education prevailed. The largest part of the respondents were people addicted to opiates (*n* = 98; 56.98%), sedatives and hypnotics (*n* = 34; 19.77%), amphetamines (*n* = 30; 17.44%) and cannabis (most frequently used, *n* = 10; 5.81%). Addiction lasted approximately 3 years, and they underwent examination in the 2nd-3rd month of treatment.

The examination was anonymous and the participation was voluntary. It is worth noticing that there were no refusals. Informed consent, according to the Helsinki Declaration recommendations, was obtained from each participant and the centre management granted permission to perform the study on their patients.

### Materials

In order to examine indirect self-destructiveness and its categories, the Polish version of the “Chronic Self-Destructiveness Scale” by Kelley (CS-DS) was administered. The Polish version of the tool, as the original one, is characterised by high reliability and validity, and includes: Transgression and Risk (A1), Poor Health Maintenance (A2), Personal and Social Neglects (A3), Lack of Planfulness (A4), and Helplessness, Passiveness in the Face of Problems/Difficulties (A5) categories, whose scores are summed up to provide one total indirect self-destructiveness score. Scores up to 103 are considered low, 104–160 is the range of medium scores, while scores above 160 are regarded as high [2, 4].

### Statistical Analysis

The statistical analysis of received scores applied descriptive methods and statistical inference methods. In order to describe mean values for quantitative traits, arithmetic means (M) were calculated, while the standard deviation (SD) was assumed to be the dispersion measure. The conformity of quantitative traits’ distributions with the normal distribution was evaluated by using the Shapiro-Wilk test. Due to the lack of conformity of explored variables’ distributions with the normal distribution, the statistical processing of acquired results used the non-parametric Mann-Whitney U significance test. For all the analyses, the maximum acceptable type I error was assumed at α = 0.05. Asymptotic two-sided test probability p was calculated and *p* ≤ 0.05 was considered statistically significant. The statistical analyses were performed by means of the *Statistica PL 13.1* statistical package [53].

## Results

**Fig. 1:**
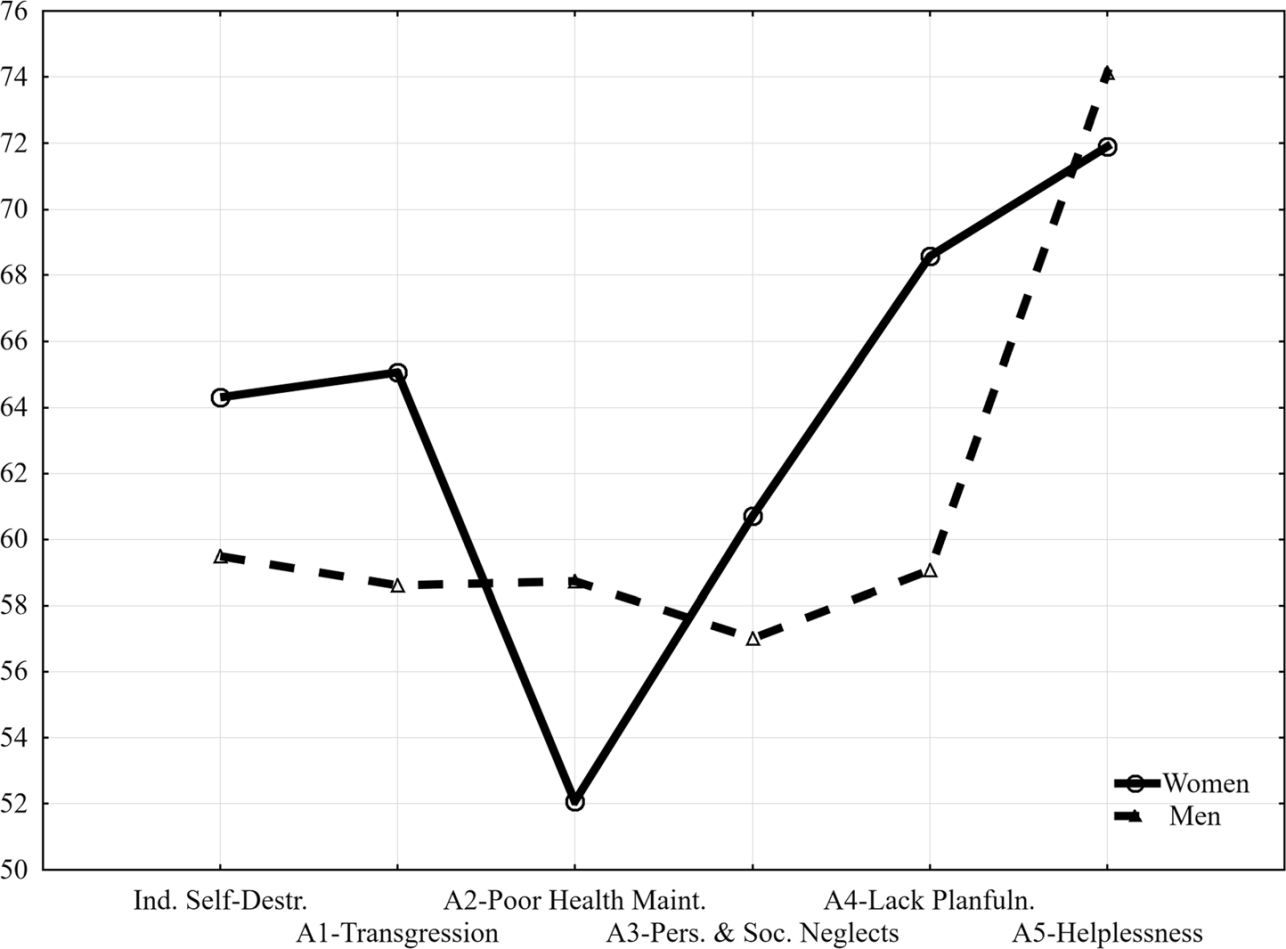
Profiles of addicted women and men in Chronic Self-Destructiveness Scale (CS-DS)

Table 1 and Fig. 1 present scores achieved on the CS-DS by addicted women and men.

**Table 1:** Gender differences between addicted women and men for the Chronic Self-Destructiveness Scale (CS-DS)

Variables	Women		Men		Significance	
	M	SD	M	SD	U	p
Indirect Self-Destructiveness	167.214	19.719	154.655	26.004	2264.000	0.001
A1-Transgression, Risk	58.571	10.498	52.759	10.748	2256.000	0.001
A2-Poor Health Maintenance	28.643	5.891	32.310	8.121	2112.000	p < 0.002
A3-Personal and Social Neglects	36.429	5.691	34.207	7.306	2568.000	0.02
A4-Lack of Planfulness	29.429	3.612	25.586	6.511	2976.000	p < 0.001
A5-Helplessness, Passiveness	7.414	1.875	10.786	18.181	2848.000	p < 0.001

The rank order of CS-DS subscales in respect of intensity for the whole study group was as follows: A5-Helplessness, A4-Lack of Planfulness, A1-Transgression and Risk, A3-Personal and Social Neglects, and A2-Poor Health Maintenance. The order was not homogenous, i.e. the same in both the whole study population and specific groups: women and men. Namely, the rank order for women was the same as that for the whole study population whereas it was different for men: A5-Helplessness, A4-Lack of Planfulness, A2-Poor Health Maintenance, A1-Transgression and Risk, and A3-Personal and Social Neglects.

Women treated for drug addiction achieved significantly higher scores on indirect self-destructiveness (general score) and subscales: A1-Transgression and Risk, A3-Personal and Social Neglects, and A4-Lack of Planfulness; and significantly lower scores on subscales A2-Poor Health Maintenance and A5-Helplessness.

Such a result is untypical as men more often achieve higher scores in other groups: in the general population [10] or in the population of individuals after suicide attempts [54].

## Discussion

The configuration of CS-DS subscales in the whole study population of addicted individuals in treatment was the same as the rank order of the subscales in women. Thus, it can be inferred that the picture and structure of indirect self-destructiveness were shaped by women who “gave them the tone”.

Differences in the rank order of CS-DS subscales are interesting since they were similar in another population of individuals experiencing problems/difficulties in psychological functioning, i.e. in the population of individuals after suicide attempts. Also in that case, women created the picture and gave the tone to indirect self-destructiveness in the whole population [54]. On the other hand, in the general population, i.e. in the population not experiencing problems/difficulties in psychological functioning, the rank order was homogenous, i.e. the same for the whole population, the group of women and the group of men [10].

Indirect self-destructiveness as a generalised behavioural tendency occurred with higher intensity in women than in men treated for drug addiction. Therefore, it can be assumed that women more frequently and/or intensely displayed tendencies and behaviours that, although convenient or pleasant at the time, may prove (physically or psychologically) harmful in the long run. It is an interesting phenomenon since indirect self-destructiveness in women was less intense than in men in the general population [10] or as intense as in men in individuals after suicide attempts [54]. Thus, it can be assumed that factors making women become addicted to drugs, or possibly addiction itself, resulted in indirect self-destructiveness as a generalised behavioural tendency being stronger in them than in men. It is worth noticing that addicted women often have life paths that are more difficult than men’s: they have been more often the victims of violence and abuse [55–57]. This may mean that they use the products to relieve suffering.

The higher intensity of indirect self-destructiveness in women than in men treated for drug addiction may be an expression of deeper psychopathology, worse adaptation and psychological and social functioning as well as a greater burden of problems and social condemnation. Various studies report more severe consequences of addiction in women than in men; those can be health, economic, legal, family, social or professional ones [58–60]. Other authors report more serious psychopathological symptoms, e.g. anxiety and mood disorders [46, 61–63].

A category of self-destructive behaviours for which women gained higher scores were transgressive and risky behaviours (A1), i.e. behaviours that violate social norms and may lead to a real threat. Drug use epitomises that type of behaviours, reflecting all aspects of that category of indirectly self-destructive behaviours: impulsive behaviours, succumbing to temptations and dependence or addiction. On the one hand, they are behaviours that transgress certain social norms (drugs). On the other hand, they are behaviours that contain a certain degree of risk resulting from the essence of using such substances, but also from behaviours undertaken under the influence of those substances.

A little more attention should be given to the issue of personal and social neglects (A3). Addicted women more frequently experience personal and social failures due to abandoning actions that might improve their personal and social situation or their interpersonal relations [4]. The higher intensity of that category in women occurred despite the attitude of caring “for everyone and everything” that is typical of women. After all, it is women who take the most care, for instance, of a child from his or her birth (at least at the beginning of the child’s life) and are also brought up in the spirit of “caring” before they become mothers [54, 64–71].

Addicted women more often report family problems and problems at home (e.g. at least one dependant), problems in social life as well as physical and mental health problems. In turn, men more commonly report greater financial problems and problems connected with crime [35, 72–74].

In many societies, women are primary caregivers, which may adversely affect their health and productivity, as the scope of their responsibility and duties usually comprises their whole extended family [65].

Another category of indirectly self-destructive behaviours, lack of planfulness (A4), may be connected with adverse events seemingly not related to the individual’s behaviour. Planning is a type of action to a great extent dependent on cognitive and motivational processes, in respect of which (according to many authors) dysfunctions occur in addicted individuals (e.g. amotivational syndrome) [75]. On the other hand, desire to use and seek a drug absorbs most of addicted women’s psychological resources, so they may no longer be sufficient for a planned and organised action.

It is worth noticing the low intensity of poor health maintenance (A2) on which women achieved the lowest score. That category contains, among others, disregarding physician’s instructions and recommendations as to coping with specific complaints as well as failure to take actions related to disease prevention, which may ultimately contribute to the worsening of symptoms and signs or even death. Poor health maintenance manifestations also include premature discontinuation of treatment, tendency to forget about appointments or procedures, as well as irregular taking of medications or giving that up completely, which is prominent in men. Other studies produced similar results, i.e. men are more prone to avoiding regular contact with physicians, while women find it more difficult to do that, irrespective of their condition as, for instance, numerous contraceptives are available only if prescribed. Besides women are more “accustomed” to using health care and more “trained” in that if only due to their necessary regular gynaecological check-ups [25, 76, 77]. Furthermore, women more frequently and willingly seek help in the case of health, life, and/or psychological problems than men who often consider such behaviour to be “unmanly” and signify weakness [37, 78]. Thus, those neglects by men have graver health consequences, which is reflected, among others, in the fact that even after suicide attempts they require more intense care than women and those attempts more frequently lead to death [37, 79]. The traditional model of masculinity is connected with less interest in positive health behaviours as such efforts are perceived as feminine, hence rejected by “real” men [41, 80].

As we could see, women more often than men use legal psychoactive substances (medicines) prescribed by physicians [29–33, 36]. The fact that women more commonly/in larger amounts use such substances is an indicator of greater care for health and/or less health neglect. Firstly, it is the physician who decides and does (or does not) prescribe the substance. Secondly, illegal psychoactive substances (which men use more often/in larger amounts) certainly do more and greater harm than medicines prescribed by physicians. It is also possible that women prefer legally obtained psychoactive substances in fear of legal (and other) consequences of obtaining/using illegal substances.

On the other hand, such a result, i.e. less health neglect in women treated for drug addiction, may be an expression of at least minimal care for their own health since they found themselves in a place where health was to be restored to them and/or they were to regain that. Anyway, studies indicate that women more often follow a regime imposed during treatment [81].

Comfortingly, women neglect their health to a significantly smaller extent than men, which holds promise for better chances of their recovery than that of men. The tendency is opposite to the one in the population of individuals after suicide attempts: women after suicide attempts neglected their health much more than men [54].

Women also ranked lower than men on helplessness (A5). Attention ought to be drawn to results of other studies indicating a relationship between indirect self-destructiveness and a sense of impotence and hopelessness [82]. In fact, as recently established, addicted men were lonely and had an unstable situation in life. Τhey were homeless and their accommodation was provided by social services [35]. It should be emphasised that helplessness in addicted women is lower than in men despite more serious problems and difficulties they encounter in their illness.

Differences in experiences connected with treatment, therapy and treatment history lie in the fact that women are more often taken care of by the health system and undergo outpatient treatment, whereas men’s treatment more commonly relies on social services and impatient facilities, including detoxification. Women more often have contact with mental health services from which they are referred for addiction treatment, while men more commonly have contact with the criminal justice system, social services and compulsory treatment [35].

When attempting to explain the higher intensity of indirect self-destructiveness in addicted women, it should be borne in mind, along with biological factors, that women encounter greater social disapproval when they use psychoactive substances [48, 83–85].

Taking psychoactive substances for recreational purposes or pleasure is generally more socially accepted in men than in women. Women taking psychoactive substances are considered especially deviant as they have violated their maternal role [44, 86, 87].

It is even believed that abusing and addicted men are easier to treat as their use is more tolerated by the society and the society blames men less than women for using e.g. alcohol [35, 44, 88].

It has been a priori assumed that psychoactive substance abuse and addiction are a problem of mainly and/or solely men and thus a great majority (or almost all) of treatment strategies have been aimed at men and taken into consideration men’s biology and psychology, generalising their results and conclusions onto the population of women too [33, 35, 48].

As we have earlier seen, many studies proved more serious harmfulness of psychoactive substance use in women. However, even side effects of the use of substances and medicines taken for therapeutic purposes are considerably more common and severe in women than in men. That concerns physical and mental complaints, symptoms and signs [89–93]. Perhaps side effect of medical treatment caused indirect self-destructiveness to be stronger in the study group of women than in men.

As far as psychopathology of addicted individuals, while externalising psychopathology is mainly found in men, women manifest both externalising and internalising symptoms [33, 94, 95]. Thus, treatment (psycho- and pharmacotherapy) should also be different. However, as findings in men are automatically “transferred” also onto women, their psychological functioning does not improve as fast, which may be manifested by higher indirect self-destructiveness in addicted women in treatment.

## Conclusions

Higher indirect self-destructiveness in addicted women may arise from the fact that a majority of addict treatment programmes is aimed at men and does not consider (or poorly considers) the specificity of women’s biology and psychology.

Moreover, that higher intensity may result from or be an expression of internalising social and cultural condemnation, which may take the form of self-blame. Maybe that is why there is a need for specific, gender-adjusted manners of intervention and treatment in addicted women. It stems from the above that the initiation and course of psychoactive substance use/addiction, as well as treatment and recovery processes, are different in women and men. Therefore, different prophylactic and, first and foremost, therapeutic strategies ought to be implemented and pursued, adjusted to the needs determined by belonging to a given sex/gender, particularly in women. For instance, it was established that addiction treatment encompassing spirituality and a sense of coherence proved effective in addicted women [96].

The author of this work hopes that the results of this study will be helpful in prophylactic and therapeutic activities. Prognostic implications include ability to identify people at risk, while therapeutic implications comprise areas such as a sense of being selfish, worse, condemned and worthless.

Optimistically, even though women are thought to be more difficult to treat, it is women who cope and function psychologically and socially better after addiction treatment [35, 97] and care more for their health [10].

Apart from less health neglect in addicted women, it should be pointed out that they also showed less helplessness than men despite more serious problems and difficulties they experienced in their disease.

Nevertheless, because the study sample is rather small and weighted toward the inclusion of men, further study of the gender differences in the consequences of indirect (chronic) self-destructiveness in drug treatment seems to be needed.

It seems pertinent to state that differences in genetics, physiology, anatomy, psychology, sociocultural expectations and experiences between women and men lay the foundation for the conclusion that women have unique health concerns related to substance use disorders [98].

## Limitations

- The difference between the number of women and men in the study group may be a possible limitation.
- Another one possible limitation may the fact that this study concerns people who have reached care, although other groups of addicted individuals are extremely difficult to explore.

## Footnotes

1 MONAR: The first Polish non-governmental organisation focused, initially, on helping drug addicted individuals, later the homeless, those who are HIV positive or suffer from AIDS, and many other groups of people needing help. It was formally established as an association in 1981, but its origins go back to October 1978.
